# Incidence of central nervous system metastases in patients with human epidermal growth factor receptor 2-positive metastatic breast cancer treated with trastuzumab: A meta-analysis

**DOI:** 10.6061/clinics/2021/e2653

**Published:** 2021-08-05

**Authors:** Xue Bai, Xue Lin, Jin Song, Jia-han Chang, Li-li Han, Cibo Fan

**Affiliations:** Medical Department of General Surgery, The First Medical Center of Chinese PLA General Hospital, Beijing 100853, China

**Keywords:** Metastatic Breast Cancer, Central Nervous System, Brain, Metastases, Trastuzumab

## Abstract

This study aimed to estimate the incidence of central nervous system (CNS) metastases in patients with human epidermal growth factor receptor 2 (HER2)-positive metastatic breast cancer (MBC) treated with trastuzumab.

Studies were identified through a literature search of electronic databases. Random-effects meta-analyses were performed to estimate the incidence rate of CNS metastases, trastuzumab therapy duration, and time from trastuzumab therapy to CNS metastasis diagnosis. A meta-analysis of odds ratios was performed to evaluate the significance of a difference in CNS metastasis incidence between patients with and without trastuzumab treatment.

Thirty studies (8121 trastuzumab-treated and 3972 control patients) were included. The follow-up duration was 18.9 months (95% confidence interval [CI]: 13.8, 24.1). The trastuzumab treatment duration was 9.0 months (95% CI: 7.0, 11.0). The median interval between the start of trastuzumab therapy and CNS metastasis diagnosis was 12.2 months (95% CI: 9.5, 14.7). The incidence of CNS metastasis after the start of trastuzumab therapy was 22% (95% CI: 16, 27). The incidence of CNS metastases was significantly higher in trastuzumab-treated than in non-trastuzumab-treated patients (odds ratio: 1.39 [95% CI: 1.06, 1.82], *p*=0.02). The survival time from the start of the study was 23.4 months (95% CI: 19.7, 27.1) in trastuzumab-treated patients and 18.4 months (95% CI: 12.7, 24.1) in patients treated with control regimens. The survival time after the development of CNS metastases in trastuzumab-treated patients was 19.2 months (95% CI: 15.6, 25.9).

Approximately 22% of patients with HER2-positive MBC who were treated with trastuzumab developed CNS metastases. However, trastuzumab-treated patients had a longer survival than patients who were not treated with trastuzumab.

## INTRODUCTION

Breast cancer is the second leading cause of cancer-related deaths in women, with a mortality rate of 2.6% in female patients (1). It accounts for 15.3% of all new cancer cases and 7% of all cancer mortalities. It is estimated that approximately 13% of women will be diagnosed with breast cancer during their lifetime (2). Although the prognosis of breast cancer has improved, it is also associated with an increased risk of metastases in the central nervous system (CNS), especially in the brain. Among women with metastatic breast cancer (MBC), 15% to 30% develop CNS metastases, which are associated with poor survival and neurological impairment (3). Generally, CNS metastases develop late in the disease course, usually after the appearance of systemic metastases, and many cases remain underdiagnosed. Studies have shown that up to 30% of patients with breast cancer have undiagnosed CNS diseases (4).

The risk factors for the development of brain metastases include young age, high-grade tumors, negative hormone receptor status, more than four lymph node metastases, and human epidermal growth factor receptor-2 (HER2) positivity (3). Progression to the CNS is also frequent in MBC patients with *BRCA* mutations, especially *BRCA2* mutations (5). Approximately 20% of women with breast cancer have a HER2-positive status, which is associated with a poor prognosis (4). Patients with overexpressing HER2 breast cancer have an increased risk of developing CNS metastases (6).

The management of breast cancer usually involves multidisciplinary treatments, including surgical interventions, radiotherapy, radiosurgery, and systemic treatments. The use of combination drugs and development of novel delivery systems have also improved in recent years (7). Systemic therapies with cytotoxic and hormonal agents and biologically targeted therapies are the core treatment options for patients with MBC (8). Immunotherapies also have a promising role in the treatment of MBC, especially in triple-negative and HER2-positive tumors (9).

Trastuzumab is a HER2-targeted humanized monoclonal antibody. It has been found to be efficacious for HER2-positive MBC and to be beneficial even after the progression of cancer (10). Trastuzumab was approved for the treatment of HER2-positive MBC in 1998 in the United States and in 2000 in Europe. Later, it was also approved for use as an adjuvant therapy in 2006 and as a neoadjuvant therapy in 2011 (11). Trastuzumab emtansine is formed by the combination of trastuzumab linked with a cytotoxic component comprising a maytansine-derivative microtubule inhibitory agent called mertansine or DM1 (12).

Although trastuzumab treatment improves the survival outcomes of patients with breast cancer, it has been found to be associated with an increased risk of CNS metastases. Many studies have reported the incidence of CNS metastases in patients with MBC during trastuzumab treatment; however, the magnitude of the incidence substantially varies across the studies. Therefore, we performed a systematic review to identify studies reporting the incidence of CNS metastases in patients with HER2-positive MBC treated with trastuzumab and conducted meta-analyses of CNS metastasis incidence and survival rates to obtain refined estimates.

## MATERIALS AND METHODS

### Ethics

All analyses were based on previously published studies; thus, no ethical approval or patient consent was required.

### Inclusion and exclusion criteria

The inclusion criteria for the studies were as follows: (a) evaluating the outcomes of trastuzumab therapy either alone or with other therapies in HER2-positive MBC patient cohorts, (b) reporting the incidence of CNS metastases in trastuzumab-treated patients, (c) comparing the incidence of CNS metastases between trastuzumab-treated patients and patients treated with non-trastuzumab regimens, and (d) reporting the survival outcomes of patients with HER2-positive MBC treated with trastuzumab. The exclusion criteria were as follows: (a) recruiting all patients with CNS metastases at baseline, (b) reporting the CNS metastasis incidence in patients with MBC treated with more than one regimen without differentiating the outcomes of each treatment, (c) reporting baseline CNS metastases but not the incidence during the study, and (d) case reports.

### Literature search

A literature search was conducted in electronic databases (Google Scholar, Ovid, PubMed, and Science Direct). The key terms used were “breast cancer,” “carcinoma,” “metastatic,” “metastasis,” “trastuzumab,” “brain metastases,” “central nervous system metastases,” “incidence,” “progressive,” “progression,” “recurrence,” “response,” “survival,” and “efficacy.” The bibliographies of important research and review articles were also screened. The literature search encompassed original research articles published in English before October 2020.

### Data extraction, synthesis, and statistical analysis

Demographic data, study design, molecular characteristics, treatment and control regimens, follow-up duration, treatment duration, prior therapies, cancer stage, performance status, baseline CNS metastases, CNS metastasis incidence, treatment response, and survival data were extracted from the included studies. Publication bias was assessed with Begg’s test using Kendall’s rank correlation coefficients between the effect estimates and their variances.

To estimate the incidence of CNS metastases, a meta-analysis of proportions was performed with Stata software (Stata Corporation, College Station, TX, USA) using binomial data reported by the individual studies. For variance stabilization, the Freeman-Tukey double arcsine transformation of proportions was incorporated using the exact binomial method. The median overall survival times of trastuzumab-treated and control patients were pooled using a random-effects model by deriving the variance from the study sample sizes. Follow-up periods and trastuzumab treatment periods were also pooled to achieve overall estimates, which were the inverse variance weighted averages of individual study estimates.

To determine the significance of a difference between trastuzumab and control regimens in the incidence of CNS metastases, meta-analyses of odds ratios were performed. To estimate the difference in survival between trastuzumab-treated patients with and without CNS metastases, a meta-analysis of mean differences was performed. Both meta-analyses were performed with the Cochrane Review Manager software (version 5.3; Nordic Cochrane Center, Copenhagen, Denmark). Statistical heterogeneity (inconsistency of outcomes between studies) was estimated using the I^2^ index.

## RESULTS

**Figure S1:**
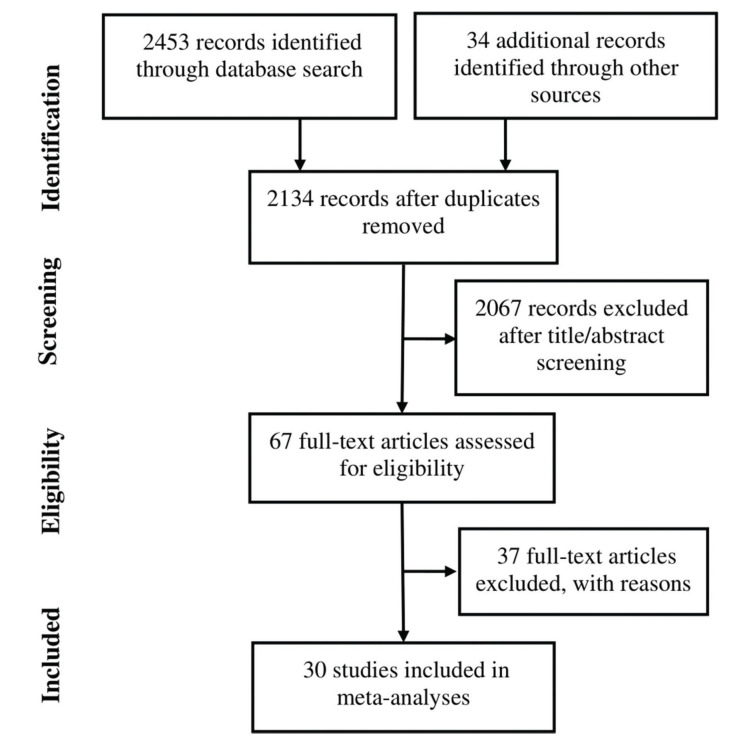
Flowchart of the screening and selection of the included studies.

**Figure S2:**
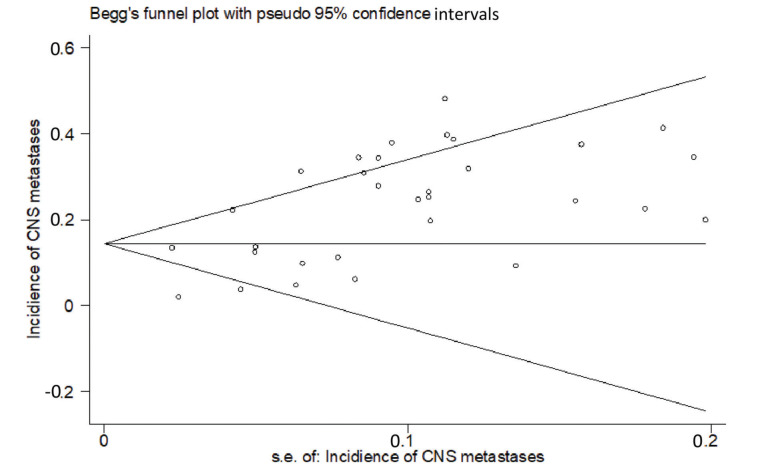
Funnel plot showing the results of Begg’s test for publication bias assessment.

Thirty studies (13-42) were included (Appendix Figure S1). In these studies, 8121 patients with HER2-positive MBC were treated with trastuzumab with or without chemotherapy, and 3972 control patients received treatments without trastuzumab. Of the included studies, 7 were randomized controlled trials, 1 was a randomized prospective study, 1 was a prospective non-randomized study, and 21 were retrospective studies. According to Begg’s test for publication bias assessment, there was no significant publication bias (adjusted Kendall’s score: 83, standard deviation: 56; *p*=0.139; Appendix Figure S2). The important characteristics of the included studies are listed in Appendix Table S1.

The percentage of patients with stage IV cancer was 32% (95% confidence interval [CI]: 13, 55). The percentage of estrogen receptor-positive patients was 47% (95% CI: 43, 51) and that of progesterone receptor-positive patients was 38% (95% CI: 32, 43). The percentage of estrogen- and progesterone receptor-positive patients was 43% (95% CI: 31, 55). Trastuzumab was used as first-line therapy in 61% (95% CI: 10, 99) of the patients.

**Figure 1:**
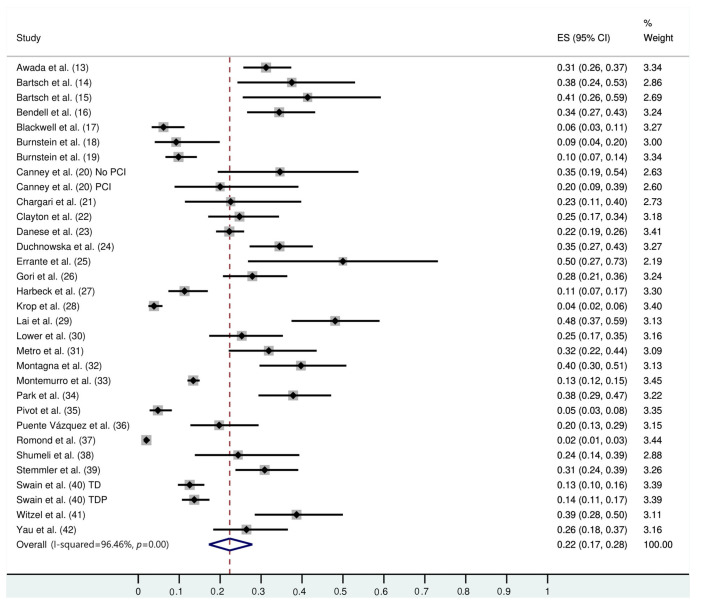
Forest graph showing the results of the meta-analysis of the incidence rate of CNS metastases in patients with HER2-positive MBC treated with trastuzumab.

**Figure 2:**
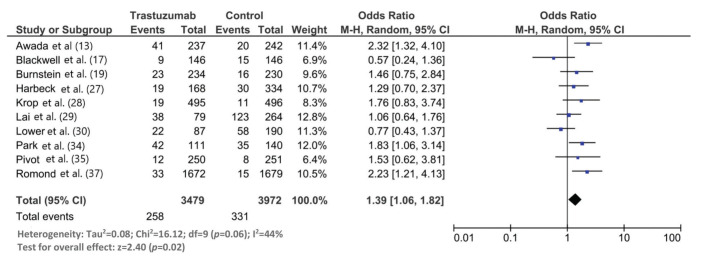
Forest graph showing the results of the meta-analysis of the incidence rate of CNS metastases in patients with HER2-positive MBC treated with and without trastuzumab.

The follow-up duration of this population was 18.9 months (95% CI: 13.8, 24.1), and the duration of trastuzumab treatment was 9.0 months (95% CI: 7.0, 11.0). The median interval between the start of trastuzumab therapy and CNS metastasis diagnosis was 12.2 months (95% CI: 9.5, 14.7). The incidence of CNS metastasis in patients with HER2-positive MBC during trastuzumab therapy was 22% (95% CI: 16, 27) (Figure 1). The incidence of CNS metastases was significantly higher in patients treated with trastuzumab-based regimens than in patients not treated with trastuzumab (odds ratio: 1.39 [95% CI: 1.06, 1.82], *p*=0.02; Figure 2).

**Figure 3:**
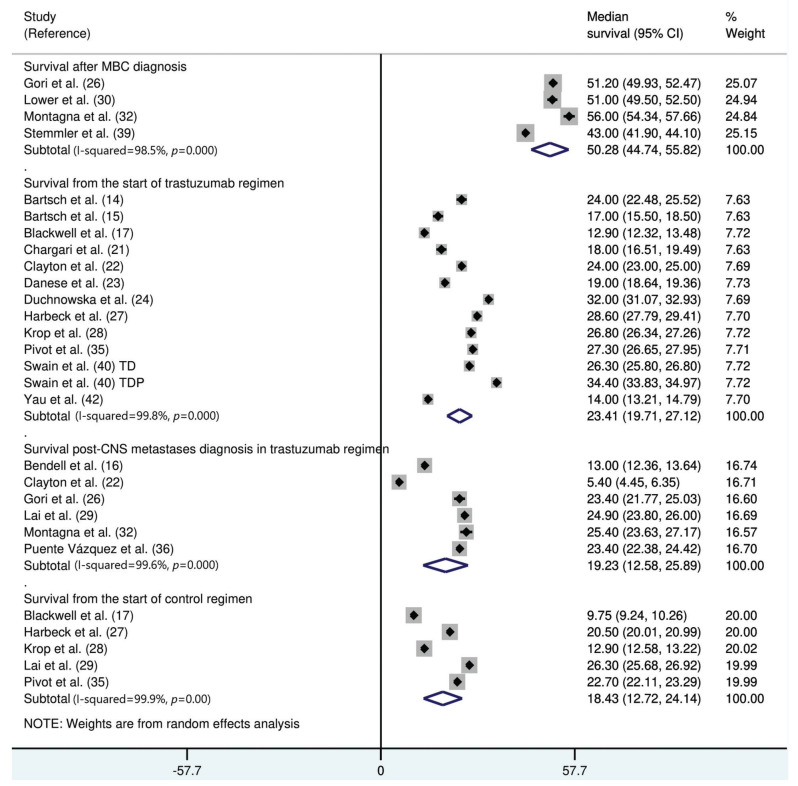
Forest graph showing the results of the meta-analysis of the median survival of patients with HER2-positive MBC treated with trastuzumab or control therapies.

**Figure S3:**
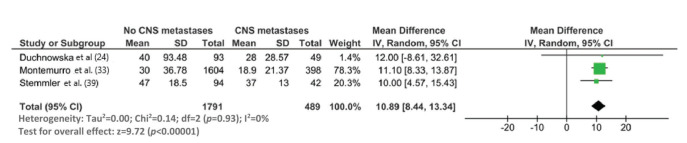
Forest graph showing the results of the meta-analysis of the survival difference between MBC patients with and without CNS metastases.

The median survival time from the start of trastuzumab therapy in patients with MBC treated with trastuzumab was 23.4 months (95% CI: 19.7, 27.1), whereas the survival time after treatment with control regimens was 18.4 months (95% CI: 12.7, 24.1). The survival time after the development of CNS metastases in trastuzumab-treated patients was 19.2 months (95% CI: 15.6, 25.9) (Figure 3). The survival time from the start of trastuzumab therapy was significantly shorter in patients with CNS metastases than in patients who did not develop CNS metastasis (mean difference: 10.9 months [95% CI: 8.4, 13.3], *p*<0.0001; Appendix Figure S3).

**Figure 4:**
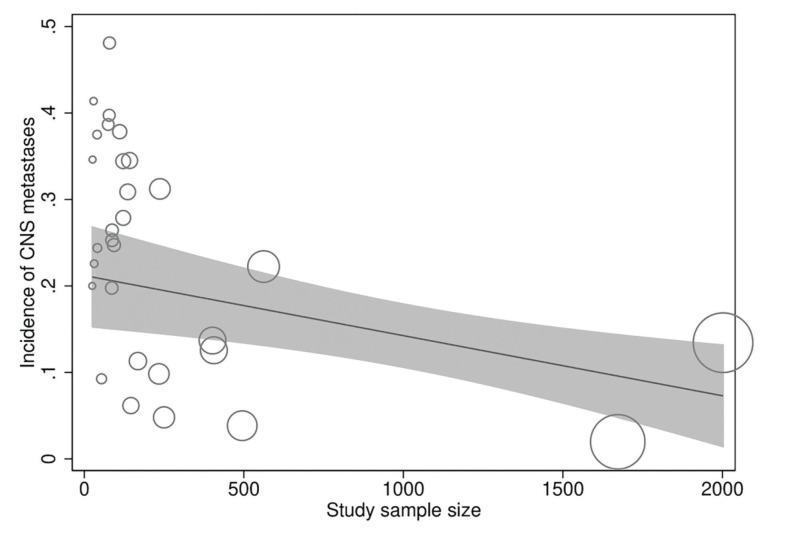
Meta-regression scatterplot showing the relationship of the incidence rate of CNS metastases in patients with HER2-positive MBC treated with trastuzumab to the study sample size.

In meta-regression analyses, the incidence rate of CNS metastases was not statistically significantly associated with study duration (meta-regression coefficient [MC]: 0.005 [95% CI:−0.003, 0.013], *p*=0.194), trastuzumab treatment duration (MC:−0.0004 [95% CI:−0.010, 0.009], *p*=0.922), age (−0.002 [95% CI:−0.012, 0.008], *p*=0.672), baseline CNS metastases (MC:−0.0004 [95% CI:−0.001, 0.0002], *p*=0.256), estrogen receptor positivity (MC: −0.006 [95% CI:−0.022, 0.010], *p*=0.438), progesterone receptor positivity (MC: 0.002 [95% CI:−0.012, 0.016], *p*=0.774), or study publication year (MC:−0.00005 [95% CI:−0.0002, 0.0001], *p*=0.367). However, the incidence rate of CNS metastases was significantly inversely associated with the study sample size (MC:−0.0001 [95% CI:−0.0002, −0.00002], *p*=0.013) independently (Figure 4) and in multivariate analyses with all of the above explanatory variables.

## DISCUSSION

In this meta-analysis, we found that approximately 22% of patients treated with trastuzumab, either alone or in combination with chemotherapy, developed CNS metastases approximately 1 year after the start of trastuzumab therapy. The duration of trastuzumab therapy was approximately 9 months. The incidence of CNS metastases was significantly higher in trastuzumab-treated patients than in control patients. The survival time from the start of trastuzumab and control therapies was approximately 23 and 18 months, respectively. The survival time after the development of CNS metastases in trastuzumab-treated patients was approximately 19 months.

In a multicenter, prospective, observational cohort study of about 1000 patients with HER2-positive MBC with trastuzumab as the predominant treatment agent, 9% of the patients had CNS metastases at MBC diagnosis and 22% additional patients developed CNS metastases after a median follow-up of 28 months (43). These outcomes provide further support to the findings of the present study. However, a considerable variability was observed in the incidence of CNS metastases (range: 2%-50%) in the included studies, and a significant inverse relationship was found between the study sample size and CNS incidence.

Krop et al. (28) found a similar incidence of CNS metastases in the trastuzumab and capecitabine groups and noted that the absolute risk of CNS progression was higher in patients with baseline metastases (28). Moreover, because the survival of patients with CNS metastases at baseline remains shorter than those without baseline CNS metastases (33), the number of patients with baseline metastases can influence the incidence rate of CNS metastases in a study. Young age and an estrogen receptor-negative status are also considered risk factors for CNS metastases (44,45).

Although we found a statistically significantly higher incidence of CNS metastases in patients with HER2-positive MBC treated with trastuzumab than in those treated with control regimens, this meta-analysis needs to be elaborated. Seven of the 10 included studies in this meta-analysis did not find a statistically significant difference in the CNS metastasis incidence between the trastuzumab and control regimens. Two studies found a higher incidence of CNS metastases with the control regimen than with the trastuzumab regimen. In this meta-analysis, the overall incidence of CNS metastases was 14% (95% CI: 7, 22) in the trastuzumab group and 12% (95% CI: 4, 22) in the control group.

Metro et al. (31) reviewed 69 patients with MBC treated with trastuzumab, of whom 22 developed CNS metastases during the treatment. Of the patients who developed CNS metastases, 10 continued trastuzumab and 10 discontinued trastuzumab to receive second-line chemotherapy and radiotherapy. The median time to progression in patients who continued trastuzumab was 10 months (95% CI: 1, 21); however, it was 7 months (95% CI: 1, 15) in patients who stopped trastuzumab (31). Swain et al. (40) found a longer time to the development of CNS metastases in pertuzumab-trastuzumab-docetaxel-treated patients than in trastuzumab-docetaxel-treated patients. Such observations support the growing evidence of the effectiveness of trastuzumab in preventing or delaying progression to the CNS.

We found that the survival time of patients (with or without CNS metastases) treated with trastuzumab was approximately 2 years, whereas the approximate survival time of patients treated with non-trastuzumab therapies was 18 months. In trastuzumab-treated patients, the survival time after the diagnosis of CNS metastasis was approximately 19 months. A difference in the survival time after CNS metastasis of as much as 14 months has been observed between patients with and without trastuzumab treatment (46). This shows that trastuzumab offers an overall benefit to patients with HER2-positive MBC despite conferring a marginally increased risk of CNS metastases. This appears to have practical implications, as evident from the continued use of trastuzumab therapy even after cancer progression in several trials (27).

This study had some limitations. The detection of a high statistical heterogeneity in some analyses is an important consideration. The wide range of CNS metastasis incidence and confounders such as age, estrogen/progesterone receptor status, and follow-up durations may have affected the outcomes. We could not perform a quality assessment of the included studies because of the varying study designs. For some variables, such as performance status, cancer stage, and progression-free survival, fewer data were available. In addition, insufficient information was available for performing subgroup analyses with respect to the combinations used in trastuzumab regimens.

## CONCLUSIONS

Approximately 22% of patients with HER2-positive MBC developed CNS metastases during trastuzumab treatment, although the range was rather wide and the incidence of CNS metastases increased with decreasing study population size, indicating that the actual incidence may be lower than that estimated in the present study. However, trastuzumab-treated patients had a longer survival than patients treated with non-trastuzumab therapies.

## AUTHOR CONTRIBUTIONS

Bai X wrote the manuscript. Lin X and Song J collected the data. Chang JH, Han LL and Fan C analyzed the data.

## APPENDIX

**Table S1:** Important characteristics of the included studies.

Study	nT	nC	Trastuzumab regimen	Control regimen	Study duration (months)	Treatment duration (months)	Age (years)	Therapy line					ER/PR status (% +)			Cancer stage				ECOG PS		
								1st	2nd	3rd	4th	5th	ER/PR	ER	PR	I	II	III	IV	0	1	2
Awada 2016	237	242	T-PAC	Neratinib-PAC	23 (IQR: 13.8-32.3)	11.5 (IQR: 5.8-18)	55 (IQR: 47-62)						52							64.1	33.33	2.11
Bartsch 2007	40		T-CAP		19 (range: 3-42)		58.5 (range: 29-73)	0	52.5	27.5	10	10		40	25	18	42.5	20	17.5			
Bartsch 2008	29		T-GEMB				60 (range: 29-83)		27.6	27.6	34.5	10				21	51.7	13.8	10.34	80-100 KPS 100%		
Bendell 2003	122		T-CT		23	7.1	50	45.1														
Blackwell 2010	146	146	T-lapatinib	Lapatinib			52 (range: 26-81)						50							58	42	5
Burnstein 2003	54		T-VINO		5.6 (range: 0.5-16+)		54.5 (range: 29-82)						37							70.4	27.78	1.852
Burnstein 2005	234	230	T-PAC/DOX/CCP	PAC/DOX/CPD	7 (95% CI: 6.4-7.3)		53 (SD: 11)	100											100	KPS 90-100 68%		
Canney 2015	51		T-taxane/PC		30.7 (range: 3.2-47.5)																	
Chargari 2011	31		T-RT		18.5 (range: 1.3-65.1)		49 (range: 36-65)							38.7		16	29	38.7	16.13			
Clayton 2004	93		T/CT			8 (range: 0-52)	48 (range: 23-76)	47.3	33.3	19.4				44.1								
Danese 2012	562		T-CT		13.9 (IQR: 6.6-23.7)	36	75 (IQR: 71-79)						25			I-III 336			40.21			
Duchnowska 2012	142		T-taxane/VINO/CAP		29 (range: 1-115)	10 (range: 1-115)	53 (range: 25-79)							38.7	30.3							
Errante 2007	14		T-CT																			
Gori 2007	122		T/CT		28 (range: 2-167)		48 (range: 28-79)	54.1	28.7	17.2				44.3	60.7							
Harbeck 2016	168	334	T-VINO	Afatinib-VINO	9.3 (IQR: 3.7-16)	4.7 (IQR: 2.1-7.4)	53.1 (SD: 12.3)							47.6	29.8					60.1	39.29	0.595
Krop 2014	495	496	T	Lapatinib-CAP	19.1		53 (range: 25-84)						57							60.4	39.19	
Lai 2004	79	264	T	No T			47.3 (SD: 10.3)							57	45.6							
Lower 2003	87	190	T-CT	CT		17.8 (range: 0.2-51)	50 (range: 29-82)							58.6	44.8	15	49.4	18.4	16.09			
Metro 2015	69		T-CT/HT																			
Montagna 2009	78		T-CT		35.3		52 (range: 26-79)						50									
Montemurro 2020	2002		T		20.6 (range: 0-50)	5.1 (range: 1-46)	54.4 (26-88)	1.35	28.3	22.3	17.9	10	62						27.32			
Park 2009	111	140	T-CT			6.4 (range: 1.2-22.5)	48 (range: 25-71)							36.9	30.6	16	27	37.8	17.12			
Povit 2015	250		T-CAP	Lapatinib-CAP										49	32				18	97		3
Puente Vázquez 2006	86		T-CT	CT			50.5 (range: 26-76)															
Romond 2005	1672	1679	T-PAC/DOX/CPD	PAC/DOX/CPD	24	13								51.6	39.2							
Shumeli 2004	41		T-VINO/taxane																			
Stemmler 2006	136		T-CT																			
Swain 2014	808		T-DOC/pertuzumab		30		53 (range: 22-80)	100					30									
Witzel 2011	75		T		24 (range: 1-98)	11 (range: 1-50)	55 (31-84)							45.3		6.7	37.3	16	30.67			
Yau 2006	87		T-CT		11	7.8 (range: 0-34)																

Abbreviations: CAP, capecitabine; CPD, cyclophosphamide; CT, chemotherapy; DOC, docetaxel; DOX, doxorubicin; ECOG PS, Eastern Ontario Cooperative Group performance status; GEMB, gemcitabine; IQR, interquartile range; PAC, paclitaxel; PCI, prophylactic cranial irradiation; RT, radiotherapy; SD, standard deviation; T, trastuzumab; VINO, vinorelbine; ER, estrogen receptor; PR, progesterone receptor; KPS, Karnofsky Performance Status.
